# Effectiveness of a cognitively grounded mathematics intervention in a high school blended learning environment: achievement gains, implementation fidelity, and demographic variability

**DOI:** 10.3389/fpsyg.2026.1800998

**Published:** 2026-08-06

**Authors:** Rusen Meylani, Ismet Kaya

**Affiliations:** Department of Educational Sciences, Ziya Gokalp Faculty of Education, Dicle University, Diyarbakir, Türkiye

**Keywords:** blended learning, cognitive learning, implementation fidelity, mastery-based instruction, mathematics achievement

## Abstract

**Introduction:**

This randomized pilot study evaluated the effectiveness of a cognitively grounded mathematics intervention implemented in a high school blended learning environment. The intervention was designed to support durable mathematical learning through mastery-based progression, retrieval-oriented practice, adaptive sequencing, structured feedback, and instructional supports. Grounded in principles from cognitive load theory, retrieval-based learning, mastery learning, adaptive instruction, and growth mindset research, the study examined whether this instructional model improved secondary mathematics achievement compared with business-as-usual mathematics instruction.

**Methods:**

Participants were 255 eleventh-grade students from a large urban public high school. Students were randomly assigned to either a Treatment group, which received the cognitively grounded blended mathematics intervention for one academic year, or a Control group, which continued with business-as-usual mathematics instruction. Mathematics achievement was measured using statewide high-stakes examinations administered before and after the intervention. Baseline equivalence between groups was examined across overall mathematics achievement and related mathematics subdomains. Outcome analyses compared posttest achievement while controlling for pretest performance, and gain-score analyses were conducted to examine growth from pretest to posttest. Additional analyses examined mathematics performance subdomains, implementation fidelity within the Treatment group, demographic sensitivity patterns, and the association between growth mindset and mathematics gains among Treatment students.

**Results:**

Baseline analyses confirmed that the Treatment and Control groups were equivalent before the intervention across overall mathematics achievement and related mathematics subdomains. After controlling for pretest performance, students in the Treatment group achieved significantly higher posttest mathematics scores than students in the Control group, with an adjusted mean advantage of approximately 5 points. Gain-score analyses supported these findings, showing greater mathematics growth and reduced outcome variability among Treatment students. Subdomain analyses indicated significant Treatment advantages in retrieval, procedural processing, conceptual application, data interpretation, and mathematics items of low, medium, and high complexity. Within the Treatment group, implementation fidelity was positively associated with mathematics achievement gains, suggesting that stronger enactment of the intervention corresponded to larger student gains. Exploratory demographic sensitivity analyses suggested that the intervention effect was broadly consistent across gender, race/ethnicity, parental education, lunch status, and living arrangement, although these findings should be interpreted cautiously because of the pilot design and unequal subgroup sizes. Growth mindset, measured only within the Treatment group, was moderately associated with mathematics gains, indicating that motivational beliefs may help explain individual differences in responsiveness to cognitively structured instruction.

**Discussion:**

Overall, the findings provide outcome-level evidence that a cognitively grounded, mastery-oriented mathematics intervention was associated with meaningful improvements in secondary mathematics achievement in the blended learning context examined. The results are consistent with theoretical expectations from cognitive load theory, retrieval-based learning, mastery learning, adaptive instruction, and growth mindset research. However, the study measured achievement outcomes and mathematics performance subdomains rather than directly measuring the cognitive and motivational mechanisms presumed to support learning. Therefore, causal claims about the mechanisms underlying the observed achievement gains should be made cautiously. Future multisite and longitudinal studies should incorporate process measures, delayed assessments, and broader samples to examine how cognitive and motivational factors contribute to learning, retention, and transfer across educational contexts.

## 1 Introduction

### 1.1 The challenge of secondary mathematics instruction

Persistent difficulties in secondary mathematics achievement constitute a major barrier to later participation in STEM fields, as mathematics performance and engagement strongly predict academic and career trajectories in STEM-related domains (Daker et al., 2021; Jamieson et al., 2021). Large-scale evidence indicates that mathematics underperformance reflects the interaction of cognitive and affective factors rather than curricular exposure alone (Foley et al., 2017; Lau et al., 2022). Meta-analytic findings further show stronger relations between fluid intelligence and mathematics than between fluid intelligence and reading, particularly for complex skills and with increasing age (Peng et al., 2019). Cross-national analyses document a robust negative association between mathematics anxiety and achievement, framing persistent underperformance as a systemic issue rather than an isolated instructional failure (Foley et al., 2017; Lau et al., 2022).

Affective barriers contribute directly to fragile mathematics learning. Research demonstrates a bidirectional relationship between mathematics anxiety and performance, with anxiety disrupting working-memory resources essential for problem solving (Carey et al., 2016; Foley et al., 2017; Lau et al., 2022). Behavioral consequences include avoidance of math-intensive coursework, which further limits access to prerequisite knowledge and reinforces long-term under-preparation (Devine et al., 2018). Longitudinal evidence indicates that mathematics anxiety predicts STEM avoidance independently of measured ability, underscoring the need for instructional designs that address anxiety-linked cognitive mechanisms (Daker et al., 2021; Jamieson et al., 2021).

Structural and contextual factors further shape secondary mathematics outcomes. Teacher qualifications and learner beliefs—such as self-efficacy, utility value, and identity—are consistently associated with achievement, indicating that instructional quality and student attitudes jointly influence performance (Rozgonjuk et al., 2020; Saenz et al., 2023). Socioeconomic context moderates relations between cognitive abilities and mathematics outcomes early in development, contributing to later disparities (Peng et al., 2019). Taken together, these findings position secondary mathematics underperformance as the product of interacting cognitive constraints, affective burdens, and contextual inequalities rather than insufficient instructional activity alone (Lau et al., 2022; Peng et al., 2019).

### 1.2 Blended learning and the limits of modality-based reform

Blended and technology-rich learning environments are frequently promoted as solutions to inefficiency and lack of differentiation; however, evidence grounded in cognitive load theory indicates that learning outcomes depend primarily on instructional design rather than delivery modality (Ginns and Leppink, 2019; Sweller et al., 2019). Cognitive load theory conceptualizes learning as constrained by limited working memory and supported by long-term memory schema acquisition, implying that instruction that amplifies extraneous load undermines retention and transfer even when engagement is high (Sweller et al., 2019). Meta-analytic evidence in multimedia learning demonstrates that reductions in total cognitive load are directly associated with improved retention and transfer, linking learning outcomes to processing demands rather than technological features (Xie et al., 2017).

Research on technology-enhanced mathematics instruction reflects this same design dependence. Reviews of virtual mathematics environments emphasize deliberate management of intrinsic and extraneous load to support learning (Hery Murtianto et al., 2022), while experimental studies show that learning effects in digital games arise from cognitive design constraints rather than gamification or digitization alone (Es-Sajjade and Paas, 2020). Measurement research further indicates substantial variability in the reliability of cognitive-load instruments, underscoring the need for careful evaluation of blended learning interventions (Krieglstein et al., 2022; Longo, 2022). Prior systematic evidence suggests that technology-enhanced mathematics instruction yields positive outcomes primarily when embedded within pedagogically coherent designs and supported by teacher preparedness and institutional capacity (Meylani, 2025a). Related work on mobile learning in mathematics similarly emphasizes that digital tools can expand access, flexibility, engagement, and differentiated practice, but that their effectiveness depends on instructional integration, teacher readiness, learner support, and attention to implementation challenges rather than on mobile access alone (Meylani, 2025b). Collectively, this literature supports interpreting blended learning as an enabling structure whose effectiveness depends on cognitively grounded design rather than modality itself (Ginns and Leppink, 2019; Sweller et al., 2019; Xie et al., 2017).

### 1.3 Cognitive science as a foundation for instructional design

Cognitive science provides a principled account of why durable mathematics learning depends on instructional structure rather than exposure or pacing. Cognitive load theory synthesizes evidence showing that working-memory limitations constrain novel problem solving and that instruction supports learning by promoting schema acquisition in long-term memory (Ginns and Leppink, 2019; Sweller et al., 2019). Contemporary syntheses emphasize distinguishing intrinsic, extraneous, and germane load and treating instruction as a mechanism for managing these components in alignment with learner expertise (Krieglstein et al., 2022).

Although the present study focuses on secondary mathematics, the broader mathematics-learning literature indicates that mathematical thinking develops through structured opportunities to reason, represent, classify, compare, generalize, and connect symbolic and informal understandings across developmental periods (Meylani, 2024b). This perspective supports the present study’s assumption that durable mathematics learning depends not merely on repeated exposure to content, but on instructional designs that organize cognitive activity, support conceptual connections, and stabilize mathematical understanding over time.

Empirical evidence identifies design features that strengthen retention and transfer in mathematics-relevant contexts. Reductions in total cognitive load predict improved retention and transfer (Xie et al., 2017), while worked examples and problem completion tasks constrain unproductive search and support schema construction (Es-Sajjade and Paas, 2020; Ginns and Leppink, 2019; Wu et al., 2017). Research further demonstrates that cognitively supportive designs buffer the negative effects of mathematics anxiety on learning and engagement, indicating that instructional structure mitigates anxiety-linked resource depletion (Lau et al., 2022; Mesghina et al., 2024).

Durable learning also depends on time-structured practice that supports retrieval and reduces cognitive resource depletion (Chen et al., 2017; Sweller et al., 2019). These mechanisms are particularly relevant in secondary mathematics, where transfer requires discrimination among problem types under cognitive constraints (Berkowitz and Stern, 2018; Peng et al., 2019). Collectively, this body of work supports instructional designs that integrate guidance, structured progression, and transfer-oriented practice to stabilize learning and reduce cognitive and affective barriers (Lau et al., 2022; Mesghina et al., 2024; Sweller et al., 2019; Wu et al., 2017; Xie et al., 2017).

### 1.4 Purpose of the study and research questions

The purpose of this study is to evaluate the effectiveness of a cognitively grounded mathematics intervention implemented within a high school blended learning environment. The intervention was designed around mastery learning, retrieval-based practice, adaptive sequencing, and structured instructional supports. The study examined whether students receiving this instructional model demonstrated greater mathematics achievement than students receiving business-as-usual instruction over a full academic year.

From a psychological and learning-sciences perspective, the intervention was derived from cognitive learning theory rather than from a modality-based assumption that blended learning is inherently superior to traditional instruction. Cognitive load theory and retrieval-based learning frameworks suggest that structured progression, repeated retrieval opportunities, feedback, and adaptive sequencing may support learning by organizing instructional conditions around known constraints of learning and memory. However, the present study did not directly measure cognitive load, working-memory demands, retrieval success, schema acquisition, or related cognitive processes. Therefore, the study evaluates educational outcomes associated with a theoretically grounded instructional model rather than directly testing the cognitive mechanisms proposed by the framework.

Although blended learning, mastery learning, and retrieval-based practice have each been widely studied, fewer randomized studies have examined a blended mathematics intervention that integrates mastery-based progression, retrieval-oriented practice, adaptive sequencing, and implementation supports within an authentic high school setting. This gap motivated the present investigation.

The study is guided by the following research questions:

1 Does participation in a cognitively grounded blended mathematics intervention result in higher posttest mathematics achievement, after controlling for baseline achievement, than business-as-usual instruction?
2 As a supplementary robustness check, do students receiving the intervention demonstrate greater mathematics achievement gains than students receiving business-as-usual instruction?
3 To what extent is implementation fidelity associated with student mathematics achievement gains within the Treatment group?
4 Does the intervention effect vary across demographic subgroups after controlling for baseline achievement?

By addressing these questions, the study evaluates whether an instructional model derived from cognitive learning theory is associated with improved mathematics achievement and more consistent learning outcomes within the educational context examined. Because cognitive processes were not directly measured, interpretations regarding cognitive mechanisms remain theoretical and should be understood as explanations consistent with the intervention design rather than as empirically verified mediating processes.

## 2 Literature review

### 2.1 Instructional structure, mastery, and mathematics achievement

Research grounded in cognitive architecture conceptualizes mathematics learning as the accumulation of long-term memory schemas that reduce working-memory demands during complex problem solving, positioning instructional structure as a primary determinant of learning outcomes rather than a peripheral delivery feature (Glaze et al., 2021; Nye et al., 2018). Cognitive load theory (CLT) specifies that instruction failing to manage intrinsic and extraneous load results in inefficient processing and weaker schema acquisition, particularly for novel or complex material (Glaze et al., 2021; Villegas-Ch et al., 2025). In mathematics, where performance relies heavily on higher-order cognitive resources, meta-analytic evidence demonstrates stronger relations between fluid intelligence and mathematics than reading—especially for complex skills—underscoring the importance of structured progressions that stabilize prerequisite competencies before advanced learning (Villegas-Ch et al., 2025). Predictors of advanced STEM achievement similarly highlight the role of domain-relevant competencies beyond general intelligence, reinforcing the need for instructional sequences that secure foundational knowledge and reduce cumulative fragility (Glaze et al., 2021; Villegas-Ch et al., 2025).

CLT-informed design research demonstrates that unit-by-unit sequencing and mastery-oriented progressions reduce cognitive load and align with worked example and problem completion effects, supporting stable understanding before advancement (Glaze et al., 2021; Nye et al., 2018). Syntheses of CLT research further describe instructional designs that mitigate obstructions to learning—such as split attention—by emphasizing structured guidance rather than pacing or exposure alone (Glaze et al., 2021; Nye et al., 2018). Evidence also indicates that structured instructional supports moderate the negative association between mathematics anxiety and learning, suggesting that mastery-oriented designs stabilize performance when affective factors tax cognitive resources (Bernacki and Walkington, 2018; Mastorodimos and Chatzichristofis, 2019). Because mathematics anxiety compromises cognitive resources essential for problem solving, instructional structures that reduce extraneous processing demands support more stable learning trajectories and limit cumulative gaps (Bernacki and Walkington, 2018; Mastorodimos and Chatzichristofis, 2019).

Instructional structure also interacts with broader sources of variability in secondary mathematics contexts. Research links students’ beliefs (e.g., utility value, self-efficacy, identity), attitudes, and teacher qualifications to mathematics achievement, indicating that enacted instructional structure and learner dispositions jointly shape outcomes (Martin et al., 2021; Mastorodimos and Chatzichristofis, 2019). Cross-national analyses disentangle individual- and contextual-level effects of mathematics anxiety on achievement, reinforcing that disparities reflect both learner-level and system-level influences (Bernacki and Walkington, 2018; Mastorodimos and Chatzichristofis, 2019). Longitudinal evidence further shows that mathematics anxiety predicts STEM avoidance and underperformance independently of ability, suggesting that secondary instructional structures permitting unstable learning and persistent anxiety contribute to downstream stratification in advanced pathways (Daker et al., 2021; Jamieson et al., 2021). Collectively, this literature supports mastery-oriented structuring as a mechanism that operationalizes cognitive constraints and mitigates anxiety-linked interference, whereas loosely structured progression amplifies variance through prerequisite instability (Bernacki and Walkington, 2018; Glaze et al., 2021; Mastorodimos and Chatzichristofis, 2019; Nye et al., 2018).

### 2.2 Retrieval-based practice and durable mathematical learning

Durable mathematics learning is supported primarily through cognitively managed practice, distributed practice (spacing), and transfer-oriented processing rather than exposure-based pacing (Glaze et al., 2021; Villegas-Ch et al., 2025). Within CLT, durable learning reflects schema construction and automation in long-term memory, with instructional activities evaluated by their effects on intrinsic and extraneous load during problem solving (Glaze et al., 2021; Villegas-Ch et al., 2025). Practice that requires learners to generate solutions engages schema-building processes, while worked examples and problem completion tasks reduce unproductive search and allocate cognitive resources toward understanding problem structures (Glaze et al., 2021; Nye et al., 2018). Empirical evidence that worked examples buffer the negative effects of mathematics anxiety further indicates that structured practice preserves cognitive resources under affective load (Bernacki and Walkington, 2018; Mastorodimos and Chatzichristofis, 2019).

Extensions of CLT incorporating resource depletion emphasize the role of spacing and interleaving as structural properties of practice that improve learning through cognitive-resource dynamics rather than repetition alone (Glaze et al., 2021; Villegas-Ch et al., 2025). Evidence shows that varying problem types benefits learning relative to blocked practice, supporting instructional designs that emphasize discrimination among problem categories and reduce reliance on superficial similarities (Villegas-Ch et al., 2025). These findings align with CLT’s emphasis on managing intrinsic load through sequencing and guidance while minimizing extraneous load (Glaze et al., 2021; Villegas-Ch et al., 2025).

Durable learning is similarly reflected in multimedia research treating cognitive load reduction as a predictor of retention and transfer. Meta-analytic evidence shows that reductions in total cognitive load are associated with improved retention and transfer, indicating that durable performance depends on design features that guide attention and reduce extraneous processing (Glaze et al., 2021; Villegas-Ch et al., 2025). Related research on instructional videos, mathematics games, and AI-generated multimodal materials demonstrates that learning outcomes depend on alignment between representational design and cognitive processing demands rather than modality or interactivity alone (Glaze et al., 2021; Villegas-Ch et al., 2025).

### 2.3 Adaptive instruction, personalization, and fidelity of implementation

Adaptive instructional systems in mathematics operationalize performance-contingent sequencing, often through intelligent tutoring systems that adjust instruction based on learner performance and cognitive state (Glaze et al., 2021; Nye et al., 2018). Research emphasizes that adaptive feedback addresses heterogeneity in prior knowledge and content complexity, particularly in sequential STEM domains where static instruction fails to match diverse learner states (Shin, 2020; Villegas-Ch et al., 2025). Dialog-based tutoring designs and K–12 research syntheses further characterize adaptive learning as rule-governed, data-driven adjustment of learning paths rather than static differentiation (Chellanthara Jose et al., 2024; Glaze et al., 2021; Nye et al., 2018; Shin, 2020). These structures align with CLT by reducing mismatches between task demands and learner expertise that elevate extraneous or unmanageable intrinsic load (Glaze et al., 2021; Villegas-Ch et al., 2025).

As adaptive and AI-supported educational systems become increasingly embedded in mathematics instruction, explainability has also become central to evaluating whether system recommendations, feedback, and learner classifications are pedagogically interpretable and ethically defensible. Recent work on educational artificial intelligence emphasizes that explainability is not simply a technical feature, but a condition for transparency, trust, accountability, and responsible instructional decision-making in AI-supported learning environments (Meylani, 2025c). This concern is particularly relevant to adaptive mathematics systems, where opaque sequencing or feedback decisions may limit teachers’ ability to diagnose misconceptions and align support with learner needs.

Personalization also intersects with motivation and affect. Research links contextual personalization to situational interest and learning outcomes, while adaptability during extended online learning predicts self-efficacy and subsequent mathematics achievement (Bernacki and Walkington, 2018; Martin et al., 2021). Affective tutoring systems further demonstrate that adapting instruction to emotional states addresses mathematics anxiety and related barriers to performance (Bernacki and Walkington, 2018; Martin et al., 2021; Mastorodimos and Chatzichristofis, 2019). Because mathematics anxiety predicts STEM avoidance independently of ability, adaptive systems integrating cognitive-state and affect-sensitive mechanisms directly address persistent barriers beyond skill differences (Glaze et al., 2021; Villegas-Ch et al., 2025; Mastorodimos and Chatzichristofis, 2019).

Finally, the effectiveness of adaptive and mastery-oriented interventions depends on fidelity to the cognitive mechanisms specified by learning theory. CLT-focused reviews highlight that misconceptions about cognitive load and weak measurement practices undermine proper application, making implementation quality and theoretical alignment critical for observed outcomes (Glaze et al., 2021; Villegas-Ch et al., 2025). Measurement research documenting variability in the reliability of cognitive-load instruments further underscores the need for theory-consistent evaluation (Nye et al., 2018; Villegas-Ch et al., 2025). Teacher enactment also shapes fidelity, as educators’ conceptions influence how adaptive systems are implemented in practice (Glaze et al., 2021; Martin et al., 2021). Together, this literature positions adaptive personalization as effective when cognitive mechanisms are preserved and when implementation and measurement remain aligned with those mechanisms (Glaze et al., 2021; Villegas-Ch et al., 2025; Mastorodimos and Chatzichristofis, 2019).

## 3 Theoretical framework

### 3.1 Cognitive architecture as the foundation for instruction

This framework conceptualizes learning as constrained by human cognitive architecture, with instruction organized around the interaction between limited working memory and long-term memory schema formation as formalized in cognitive load theory (CLT) (Paas and van Merriënboer, 2020; Sweller et al., 2019). CLT specifies that working-memory capacity during novel problem solving is highly constrained and that skilled performance depends on retrieval from long-term memory, imposing structural requirements on instructional design for complex, element-interactive content (Paas and van Merriënboer, 2020; Sweller et al., 2019). Evidence across learning environments consistently indicates that cognitive processing conditions—rather than instructional format—explain variation in learning outcomes (Müller and Wulf, 2023).

Instruction that fails to manage intrinsic and extraneous load produces fragile learning and unstable transfer by diverting limited working-memory resources toward extraneous processing rather than schema construction (Leppink and van den Heuvel, 2015; Sweller et al., 2019). CLT scholarship documents persistent misapplications, including interpretations that treat cognitive load as uniformly detrimental, which contributes to inconsistent outcomes across implementations (Krieglstein et al., 2022; Leppink and van den Heuvel, 2015). Measurement research reinforces this mechanism-level framing by demonstrating substantial variability in the reliability and validity of subjective cognitive-load instruments and the absence of widely generalizable computational models based on EEG data, underscoring the need for theory-aligned, multi-method monitoring of cognitive processes (Krieglstein et al., 2022; Longo, 2022).

### 3.2 Mastery, retrieval, and adaptive constraint

Within this framework, mastery learning functions as a stabilizing mechanism that secures prerequisite knowledge before advancement, aligning instructional progression with schema acquisition and gradual reductions in working-memory demands (Ghanbari et al., 2020; Sweller et al., 2019). CLT-informed design research emphasizes unit-by-unit organization, worked examples, and problem completion tasks as strategies that reduce cognitive load and support guided schema construction, demonstrating that mastery operates through cognitive mechanisms rather than pacing alone (Sweller et al., 2019; Wu et al., 2017). In mathematics contexts, worked examples have been shown to mediate the negative association between mathematics anxiety and learning outcomes, indicating that structured guidance reallocates cognitive resources toward diagnostically relevant problem features (Mesghina et al., 2024).

Retrieval-based learning serves as the consolidation mechanism within the framework, as durable learning depends on effortful access to long-term memory rather than repeated exposure (Nelson and Eliasz, 2022; Sweller et al., 2019). Adaptive constraint operationalizes mastery and retrieval at scale through performance-contingent sequencing that aligns instructional demands with learner states while preventing premature advancement that bypasses prerequisite schemas (Glaze et al., 2021; Villegas-Ch et al., 2025). Intelligent tutoring systems exemplify this approach by adapting mathematics instruction based on learner performance and cognitive state, reinforcing sequencing grounded in cognitive architecture rather than static pacing (Glaze et al., 2021; Nye et al., 2018). Research on dialog-based tutoring and AI-supported mathematics instruction further supports rule-governed adaptation layered with feedback and retrieval opportunities (Chellanthara Jose et al., 2024; Nye et al., 2018; Shin, 2020; Villegas-Ch et al., 2025). Work on learning diagnosis and knowledge tracing similarly emphasizes interpretability and alignment with psychometric constructs, supporting constrained adaptation over opaque personalization (Wang et al., 2023).

### 3.3 Blended learning and fidelity as enabling conditions

Blended learning is treated within this framework as an enabling infrastructure rather than a learning mechanism, as educational outcomes depend on instructional methods and cognitive processing conditions rather than delivery modality itself (Noetel et al., 2021; Sweller et al., 2019). Reviews of video-based instruction and pandemic-era online learning highlight that technology facilitates instructional methods through design, access, and feedback, while effectiveness depends on implementation quality rather than mode of delivery (Dhawan, 2020; Noetel et al., 2021; Stoian et al., 2022; Thoo et al., 2021). Research in large-class and K–12 contexts further links blended environments to flexibility, scalability, and mastery monitoring, positioning technology as infrastructure that supports iterative practice cycles rather than as an independent driver of learning (Chellanthara Jose et al., 2024; Indriati et al., 2024).

Implementation fidelity is a theoretical necessity within this framework because cognitive mechanisms engage only when instructional components are enacted as specified; deviations disrupt schema acquisition and limit transfer (Leppink and van den Heuvel, 2015; Sweller et al., 2019). Evidence from professional development trials and school-level comparisons demonstrates systematic variation in fidelity associated with learning-relevant outcomes, reinforcing enactment quality as a source of outcome variability within the same reform category (Chellanthara Jose et al., 2024; Lee et al., 2021; Pfeiffer and Landa, 2022). Measurement research further emphasizes the need for fidelity monitoring aligned with CLT constructs, given limitations in subjective cognitive-load measures and the importance of triangulating cognitive and affective indicators (Krieglstein et al., 2022; Longo, 2022). Physiological indices, including pupillometry, provide objective measures of arousal and cognitive load during mathematical tasks, supporting multi-method assessment of enactment quality in blended and adaptive learning environments (Galeano and Gredebäck, 2024; Longo, 2022).

## 4 Methodology

### 4.1 Participants and setting

Participants were 255 eleventh-grade students enrolled in a large urban public high school in the southwestern United States. The school served a socioeconomically and ethnically diverse student population. Students were randomly assigned at the individual level to either a Treatment group (*n* = 127) or a Control group (*n* = 128) as part of a year-long pilot implementation of a cognitively grounded mathematics intervention during the 2016–2017 school year. Random assignment resulted in equivalent groups at baseline with respect to pre-intervention mathematics achievement.

#### 4.1.1 Ethics statement

This study involved human participants and received institutional review board approval from the relevant institutional review board (IRB). The study followed applicable institutional and ethical standards. Informed consent was obtained from all participants, participation was voluntary, and the dataset excluded personally identifying information.

### 4.2 Research design

The study employed a randomized controlled pilot design with a pretest–posttest structure. Students in the Treatment group participated in the intervention for the full academic year, while students in the Control group received business-as-usual mathematics instruction. The primary outcome was mathematics achievement gain, defined as the difference between posttest and pretest scores.

#### 4.2.1 Description of instructional conditions

To support interpretability, replication, and theory alignment, this subsection provides an explicit comparison between the cognitively grounded intervention and business-as-usual mathematics instruction. The purpose of this comparison is not to document curricular coverage or technological features, but to specify the instructional conditions under which learning occurred and to clarify how those conditions differed across groups in ways that are theoretically meaningful from a cognitive science perspective.

Consistent with cognitive load theory and retrieval-based learning frameworks, the intervention altered the structure of learning by constraining progression, organizing practice around retrieval rather than exposure, and aligning task sequencing with learner performance and prerequisite knowledge states (Nelson and Eliasz, 2022; Paas and van Merriënboer, 2020; Sweller et al., 2019). These constraints targeted working-memory demands, schema acquisition, and consolidation processes that underlie durable learning and transfer. In contrast, business-as-usual instruction reflected conventional secondary mathematics practices characterized by time-based pacing, fixed sequencing, and variable attention to mastery or cognitive load management.

The comparative features summarized in Table 1 operationalize the theoretical distinctions articulated in the cognitive framework guiding this study. Rather than representing isolated instructional techniques, these features functioned as an integrated system in which mastery-based progression, retrieval-oriented practice, adaptive sequencing, and teacher enactment jointly shaped the cognitive conditions of learning. This comparison provides a concrete referent for interpreting intervention effects, fidelity measures, and variability in student outcomes, and it anchors subsequent analyses in clearly specified instructional contrasts rather than abstract descriptions of treatment and control conditions.

**Table 1:** Instructional features of the cognitively grounded intervention compared to business-as-usual instruction.

Instructional dimension	Cognitively grounded intervention	Business-as-usual instruction
Progression structure	Mastery-based progression; advancement contingent on demonstrated understanding	Pacing determined by curriculum calendar
Practice type	Repeated retrieval-based practice with feedback	Primarily exposure-based practice
Sequencing	Adaptive sequencing based on performance	Fixed sequence of topics
Error handling	Errors trigger additional guided practice	Errors addressed inconsistently
Cognitive load management	Explicit reduction of extraneous processing	No systematic load management
Teacher role	Coach and diagnostician	Content deliverer
Student role	Active retrieval and self-monitoring	Passive reception and practice
Technology function	Constraint-based practice and monitoring	Content delivery or supplementation

To further clarify how these instructional differences manifested at the lesson level, Appendix A provides a representative paired lesson comparison drawn from the same mathematical topic and instructional timeframe. The paired example illustrates how identical content objectives were enacted under the cognitively grounded intervention and under business-as-usual conditions, with explicit attention to progression structure, practice format, error handling, and opportunities for retrieval. This example is intended to concretize the cognitive constraints summarized in Table 1 and to provide a process-level referent for interpreting intervention effects and fidelity indicators.

### 4.3 Measures

#### 4.3.1 Mathematics achievement

*Pretest*: baseline mathematics achievement was measured using the mathematics portion of a previously administered statewide high-stakes assessment aligned with college readiness standards and structurally comparable to the ACT Mathematics test.

*Posttest*: post-intervention achievement was measured using the end-of-year statewide high-stakes mathematics assessment administered to all students.

*Math gain*: mathematics gain was operationalized as the difference between posttest and pretest scores.

#### 4.3.2 Intervention fidelity measures

To capture the degree to which the intervention was implemented as intended, multiple fidelity indicators were calculated for students in the Treatment group. Fidelity was conceptualized as a continuous construct, reflecting proportional exposure and enactment rather than dichotomous participation.

##### 4.3.2.1 Professional development (PD) fidelity

Teachers assigned to the Treatment group were offered up to 10 h of professional development focused on the cognitive and instructional principles underlying the intervention. PD fidelity was calculated as the proportion of available professional development hours completed, capped at a maximum value of 1.0:

$$\mathrm{PD}\;\text{Fidelity}=\min(\frac{\mathrm{PD}\;\text{Hours Completed}}{10},1)$$

This approach ensured that PD fidelity reflected degree of exposure relative to the intended dosage, while preventing overrepresentation of participation beyond the planned maximum.

##### 4.3.2.2 Consultation fidelity

Each student in the Treatment group was eligible for up to 16 h of individualized consultation with their mathematics teacher over the academic year. Teachers maintained records of consultation hours for each student. Consultation fidelity was calculated as:

$$\text{Consultation Fidelity}=\min(\frac{\text{Consultation Hours}}{16},1)$$

This metric represented the proportion of available consultation support utilized by each student.

##### 4.3.2.3 Usage fidelity

Students in the Treatment group were provided access to the online instructional system, with a maximum intended usage of **36 h** across the academic year. Usage fidelity was computed as:

$$\text{Usage Fidelity}=\min(\frac{\text{System Usage Hours}}{36},1)$$

Capping usage fidelity at 1.0 ensured that the measure reflected adherence to the intended dosage rather than absolute time-on-task.

##### 4.3.2.4 Implementation fidelity (composite)

Overall implementation fidelity was operationalized as a weighted composite of the three fidelity components, reflecting their theoretical importance to the intervention model. Specifically, professional development fidelity and usage fidelity were each weighted at 0.40, while consultation fidelity was weighted at 0.20:

$$\begin{array}{l} \text{Implementation Fidelity}=(0.40\times \mathrm{PD}\;\text{Fidelity})\\ +(0.40\times \text{Usage Fidelity})+(0.20\times \text{Consultation Fidelity}) \end{array}$$

These weights were selected *a priori* to reflect the centrality of teacher preparation and sustained student engagement with the instructional system, while recognizing consultation as a supplemental support mechanism constrained by logistical factors (e.g., teacher caseloads).

Because the fidelity components were expected to reflect interrelated aspects of enactment quality, the composite score was treated as an implementation index rather than as a psychometric scale composed of empirically independent subdimensions. The individual fidelity indicators were therefore interpreted descriptively, and no model was used to estimate their unique independent effects simultaneously.

##### 4.3.2.5 Cognitive performance subdomains

In addition to overall mathematics achievement, the assessment was designed to generate scores reflecting distinct cognitive-performance domains aligned with the intervention’s theoretical framework. Item classifications were established during test development and were used to compute subscale scores representing retrieval, procedural processing, conceptual application, and data interpretation. Assessment items were also classified in accordance with cognitive complexity.

- Retrieval: retrieval items assessed students’ ability to recall previously learned mathematical facts, procedures, and relationships from long-term memory with minimal external support.
- Procedural processing: procedural-processing items assessed students’ ability to execute mathematical procedures accurately and efficiently, including multi-step computational operations and algorithmic problem solving.
- Conceptual application: conceptual-application items assessed students’ ability to apply mathematical concepts to novel situations, transfer previously learned knowledge, and demonstrate conceptual understanding beyond routine procedural execution.
- Data interpretation: data-interpretation items assessed students’ ability to analyze quantitative information, interpret representations, identify patterns, and draw mathematically supported conclusions from presented data.
- Complexity levels: assessment items were additionally classified according to cognitive complexity as follows:
  ◦ Low complexity: low-complexity items required straightforward recall or application of a single procedure.
  ◦ Medium complexity: medium-complexity items required integration of multiple steps or relationships and moderate reasoning demands.
  ◦ High complexity: high-complexity items required extended reasoning, conceptual integration, problem solving, or the coordination of multiple mathematical representations.

#### 4.3.3 Growth mindset

To examine individual differences associated with student learning outcomes within the intervention condition, growth mindset was assessed among students in the Treatment group. Growth mindset refers to the belief that intellectual ability and academic competence can be developed through effort, effective strategies, and learning experiences (Dweck and Leggett, 1988; Yeager and Dweck, 2020).

Students completed a self-report mindset instrument designed to assess beliefs regarding the malleability of intelligence and academic ability. Responses were aggregated to create a composite MindsetScore, with higher scores indicating stronger endorsement of growth-oriented beliefs.

Internal consistency reliability of the mindset measure was evaluated using McDonald’s omega (*ω*), which is recommended for multidimensional educational and psychological measures because it does not require the assumption of equal item loadings. Reliability was satisfactory [*ω* = 0.809, 95% CI (0.767, 0.845)]. For comparison, Cronbach’s alpha yielded a similar estimate [*α* = 0.807, 95% CI (0.762, 0.841)], indicating acceptable internal consistency.

Because mindset was measured only within the Treatment group, analyses involving mindset were exploratory and were intended to examine variability in student outcomes within the intervention condition rather than to evaluate treatment effects or moderation across instructional groups.

### 4.4 Analytic strategy

The analytic strategy was designed to evaluate intervention effects on mathematics achievement growth while addressing distributional characteristics of the data and aligning statistical methods with the study’s randomized design. All analyses were conducted using SPSS (Version 23), with an alpha level of 0.05.

#### 4.4.1 Baseline equivalence

Baseline equivalence between the Treatment and Control groups was evaluated using a univariate analysis of variance (ANOVA) on pretest mathematics scores. This analysis verified that random assignment produced groups with equivalent initial achievement, supporting causal interpretation of post-intervention differences.

#### 4.4.2 Primary outcome analysis: mathematics achievement growth

The primary outcome variable was mathematics achievement gain (MathGain), operationalized as the difference between posttest and pretest scores. Gain scores were selected as the primary metric because random assignment and demonstrated baseline equivalence eliminate the need for statistical adjustment and provide a direct representation of academic growth across the intervention period.

Group differences in MathGain were examined using an independent-samples comparison. Because preliminary diagnostics indicated substantial heterogeneity of variance between groups, Levene’s test was evaluated prior to inference. When the assumption of homogeneity of variance was violated, group differences were interpreted using the Welch unequal-variances t-test, which provides unbiased estimates under heteroscedastic conditions. This approach ensured robustness of inference while preserving transparency of effect estimation.

Effect magnitude was quantified using mean differences, confidence intervals, and partial eta squared derived from equivalent ANOVA formulations for comparability with educational research conventions.

#### 4.4.3 Supplementary analysis: posttest achievement controlling for pretest performance

As a complementary analysis, posttest mathematics scores were analyzed using analysis of covariance (ANCOVA), with pretest mathematics score entered as a covariate and group assignment as the fixed factor. This model assessed whether post-intervention group differences remained after adjusting for individual differences in baseline achievement.

Given the large sample size and random assignment, ANCOVA was treated as a confirmatory robustness check rather than as the primary estimator of intervention effects. Homogeneity of regression slopes was examined and confirmed prior to interpretation. Because variance heterogeneity was observed, ANCOVA results were interpreted in conjunction with gain-score analyses rather than in isolation.

The interaction between pretest mathematics score and instructional group was examined and was not statistically significant, indicating that the assumption of homogeneous regression slopes was satisfied.

Parallel ANCOVA models were also conducted for cognitive-performance subdomains and complexity-specific mathematics outcomes. In each model, the relevant posttest score was entered as the dependent variable, instructional group was entered as the fixed factor, and the corresponding pretest score was entered as the covariate.

#### 4.4.4 Exploratory subgroup analyses

To examine heterogeneity of response to the intervention, exploratory univariate analyses were conducted within the Treatment group only. Restricting these analyses to treated students avoided confounding intervention access with subgroup membership.

Separate ANOVAs examined MathGain as a function of gender, race/ethnicity, parental education, lunch status, and living arrangement. These analyses were descriptive and hypothesis-generating in nature, given unequal subgroup sizes and the pilot scope of the study. Effect sizes were reported using partial eta squared to facilitate interpretation of magnitude independent of statistical significance.

#### 4.4.5 Implementation fidelity analyses

Associations between mathematics gain and fidelity indicators were examined using Pearson correlation coefficients within the Treatment group. Fidelity measures included professional development fidelity, consultation fidelity, usage fidelity, and a weighted composite implementation fidelity index.

Given the conceptual and empirical overlap among fidelity indicators, correlations were interpreted as evidence of implementation sensitivity rather than as estimates of independent causal contributions. The composite fidelity measure was emphasized as the most theoretically representative index of enactment quality.

#### 4.4.6 Interpretive framework

All analyses were interpreted within a cognitive learning theory framework. Achievement gains and compression of outcome variability were interpreted as outcomes theoretically consistent with the intervention design and cognitive learning framework. Because cognitive processes were not directly measured, conclusions regarding underlying mechanisms remain inferential. Statistical models were selected to preserve alignment between theoretical claims, design features, and empirical inference.

### 4.5 Methodological rationale and alignment with results

The proportional and capped operationalization of fidelity indicators ensured that all fidelity measures reflected adherence to the intended intervention design rather than simple exposure or participation. The weighted composite implementation fidelity index was developed to capture the integrated nature of intervention enactment and aligned with the study’s emphasis on implementation quality as a continuous construct. The observed positive associations between fidelity and mathematics achievement gains suggest that stronger implementation of the intervention was associated with improved student outcomes. Given the substantial intercorrelations among fidelity indicators, particularly between professional development fidelity and overall implementation fidelity, these measures are best interpreted as reflecting interconnected dimensions of a broader implementation-quality construct rather than independent predictors of achievement.

Within this framework, mathematics achievement served as the primary educational outcome through which the effectiveness of the intervention was evaluated. Cognitive load theory, retrieval-based learning, and mastery-learning frameworks provide a theoretical rationale for expecting instructional designs that emphasize structured progression, repeated retrieval opportunities, feedback, and adaptive sequencing to support student learning (Nelson and Eliasz, 2022; Paas and van Merriënboer, 2020; Sweller et al., 2019). Accordingly, achievement gains and reductions in outcome variability were interpreted as educational outcomes consistent with the theoretical foundations of the intervention.

However, the present study was designed to evaluate instructional effectiveness rather than directly test cognitive mechanisms. No direct measures of cognitive load, working-memory demands, retrieval success, schema development, or related cognitive processes were collected. Consequently, although the observed achievement patterns are consistent with predictions derived from cognitive learning theory, the findings cannot directly verify the specific mechanisms responsible for the observed effects. Interpretations regarding cognitive processes therefore remain theoretical and should be understood as plausible explanations derived from the intervention design rather than empirically established mediators of achievement.

## 5 Results

### 5.1 Baseline equivalence across mathematics outcome measures

To evaluate the effectiveness of random assignment and establish baseline equivalence, a multivariate analysis of variance (MANOVA) was conducted comparing Treatment and Control groups on nine pre-intervention mathematics measures, including overall mathematics achievement, mathematics raw score, retrieval performance, procedural processing, conceptual application, data interpretation, and low-, medium-, and high-complexity mathematics outcomes.

The multivariate test indicated no statistically significant differences between groups across the combined set of pretest measures, Pillai’s Trace = 0.050, *F*(7, 247) = 1.84, *p* = 0.080, partial η^2^ = 0.050. Follow-up univariate analyses likewise revealed no significant group differences on any individual measure. Effect sizes were negligible across all outcomes (partial η^2^ = 0.000–0.008).

Descriptive statistics further demonstrated substantial similarity between groups. For example, Treatment students (*M* = 20.69, SD = 4.79) and Control students (*M* = 20.67, SD = 5.20) performed nearly identically on the pretest mathematics score. Similar patterns were observed for mathematics raw score, retrieval performance, procedural processing, conceptual application, data interpretation, and complexity-level outcomes.

**Figure 1:**
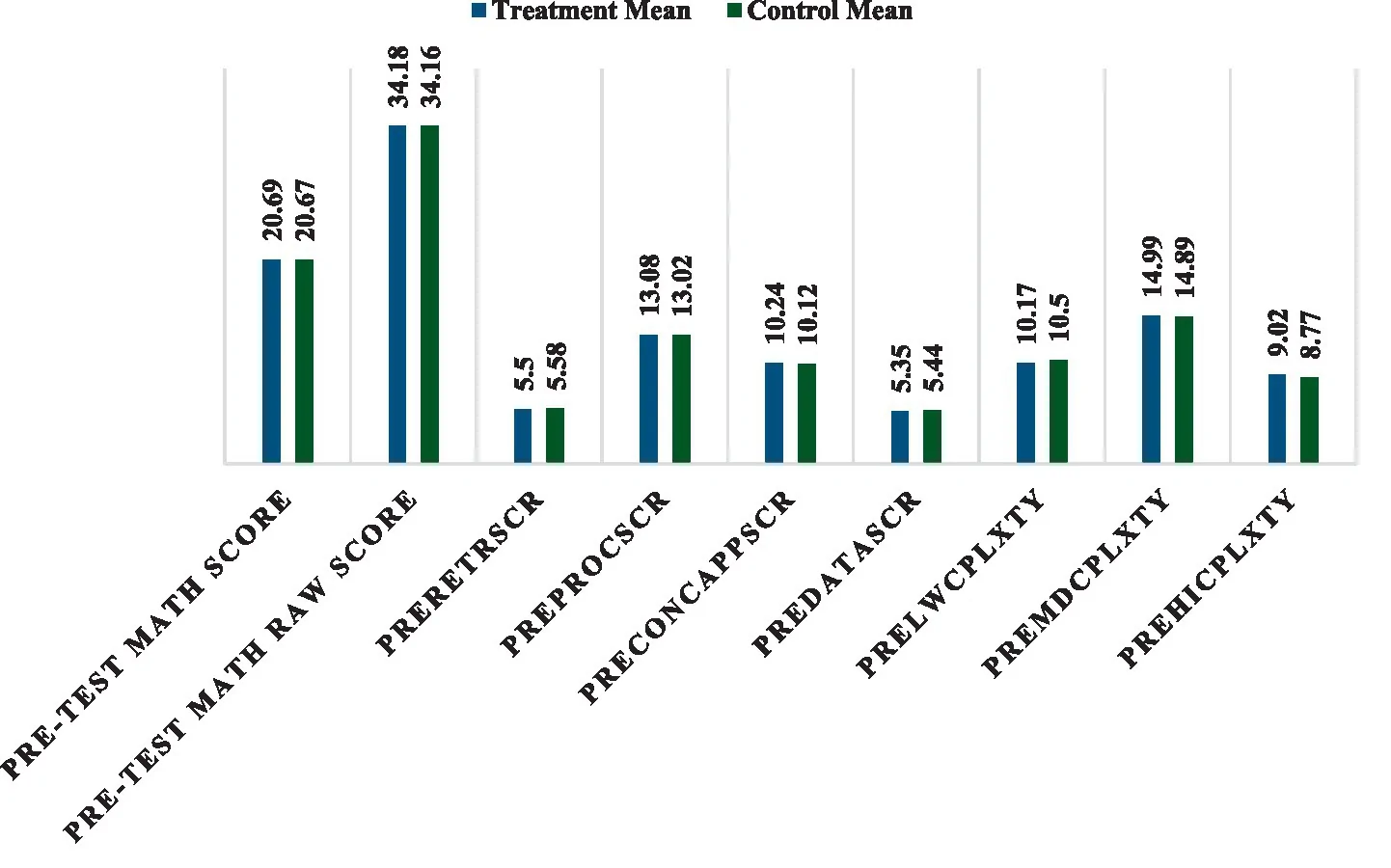
Baseline equivalence across mathematics outcome measures.

Taken together, these findings provide strong evidence of baseline equivalence and indicate that the Treatment and Control groups were statistically comparable prior to implementation of the intervention (Table 2 and Figure 1).

**Table 2:** Baseline equivalence of treatment and control groups across pretest mathematics outcomes.

Outcome measure	Treatment *M* (SD)	Control *M* (SD)	*F*	*p*	Partial η^2^
Pretest math score	20.69 (4.79)	20.67 (5.20)	0.00	0.973	0.000
Pretest math raw score	34.18 (7.99)	34.16 (8.61)	0.00	0.987	0.000
Pre retrieval score	5.50 (1.30)	5.58 (1.36)	0.20	0.656	0.001
Pre procedural score	13.08 (3.13)	13.02 (3.22)	0.02	0.890	0.000
Pre conceptual application score	10.24 (3.07)	10.13 (3.33)	0.09	0.767	0.000
Pre data interpretation score	5.35 (1.05)	5.44 (1.25)	0.33	0.566	0.001
Pre low-complexity score	10.17 (1.72)	10.50 (1.99)	1.97	0.162	0.008
Pre medium-complexity score	14.99 (3.68)	14.89 (3.96)	0.05	0.832	0.000
Pre high-complexity score	9.02 (3.08)	8.77 (3.17)	0.38	0.537	0.002

Treatment group *n* = 127; Control group *n* = 128. No statistically significant differences were observed across any pretest measure.

### 5.2 Intervention effects on mathematics achievement

#### 5.2.1 Primary analysis: posttest mathematics achievement

To evaluate the effect of the intervention on mathematics achievement while accounting for baseline performance, an analysis of covariance (ANCOVA) was conducted with posttest mathematics achievement as the dependent variable, instructional group as the fixed factor, and pretest mathematics achievement as the covariate. This approach was selected as the primary inferential analysis because it adjusts posttest performance for initial achievement differences and provides a robust estimate of treatment effects in pretest–posttest randomized designs.

The ANCOVA revealed a significant effect of pretest mathematics achievement, *F*(1, 252) = 88.21, *p* < 0.001, partial η^2^ = 0.259, indicating that baseline achievement was a significant predictor of posttest performance. After controlling for pretest achievement, instructional group remained a significant predictor of posttest mathematics achievement, *F*(1, 252) = 99.35, *p* < 0.001, partial η^2^ = 0.283 (Table 3).

**Table 3:** ANCOVA results for posttest mathematics achievement.

Source	SS	df	*F*	*p*	Partial η^2^
Pretest Mathematics Achievement	1500.72	1	88.21	< 0.001	0.259
Group (Treatment – Control)	1690.23	1	99.35	< 0.001	0.283
Error	4287.40	252	—	—	—

Estimated marginal means indicated that students in the Treatment group achieved significantly higher adjusted posttest scores [*M* = 26.73, SE = 0.37, 95% CI (26.01, 27.45)] than students in the Control group [*M* = 21.58, SE = 0.37, 95% CI (20.86, 22.30)] (Table 4).

**Table 4:** Adjusted posttest mathematics achievement by group.

Group	Adjusted mean	SE	95% CI
Treatment	26.73	0.37	[26.01, 27.45]
Control	21.58	0.37	[20.86, 22.30]

The adjusted mean difference between groups was 5.15 points, 95% CI [4.13, 6.17], *p* < 0.001. The effect size was large according to conventional benchmarks, suggesting that the intervention produced a substantial improvement in mathematics achievement beyond what could be attributed to baseline differences in student performance (Table 5).

**Table 5:** Pairwise comparison of adjusted posttest means.

Comparison	Mean difference	SE	95% CI	*p*
Treatment − Control	5.15	0.52	[4.13, 6.17]	< 0.001

Adjusted means were estimated at the grand mean of pretest mathematics achievement (*M* = 34.17). Bonferroni adjustment was applied to pairwise comparisons.

#### 5.2.2 Supplementary analysis: mathematics achievement gain

As a supplementary robustness check, mathematics achievement growth was operationalized as the difference between posttest and pretest scores (MathGain). Given individual-level random assignment and established baseline equivalence, gain scores provided a direct estimate of learning growth attributable to the intervention.

Preliminary diagnostics indicated substantial heterogeneity of variance between groups (Levene’s *F*(1, 253) = 131.96, *p* < 0.001). Given the substantial variance heterogeneity and balanced group sizes, group differences in mathematics gain were evaluated using Welch’s unequal-variances *t*-test, which provides unbiased inference under heteroscedastic conditions.

Results revealed a statistically significant and large difference in mathematics achievement growth between groups, *t*(140.69) = 8.45, *p* < 0.001. Students in the Treatment group demonstrated substantially greater gains (*M* = 6.04, SD = 1.55) than students in the Control group (*M* = 0.91, SD = 6.69). The estimated mean difference was 5.13 points, with a 95% confidence interval of [3.93, 6.33] (Table 6).

**Table 6:** Descriptive statistics and Welch’s test for mathematics gain scores.

Group	*N*	Mean	SD
Treatment	127	6.04	1.55
Control	128	0.91	6.69

Welch’s *t*(140.69) = 8.45, *p* < 0.001; Mean Difference = 5.13, 95% CI [3.93, 6.33].

In addition to higher mean gains, the Treatment group exhibited markedly reduced variability in gain scores relative to the Control group, indicating that learning gains were broadly distributed rather than concentrated among a small subset of students.

#### 5.2.3 Teacher-adjusted sensitivity analysis

To address the nested structure of the data, a teacher-adjusted sensitivity analysis was conducted with posttest mathematics achievement as the dependent variable, instructional group as the fixed factor, mathematics teacher as a random factor, and pretest mathematics raw score as the covariate. The model also included the Group × Teacher interaction to examine whether the intervention effect varied across teachers.

Descriptive statistics indicated that students in the Treatment group had higher posttest mathematics scores (*M* = 26.73, SD = 4.93) than students in the Control group (*M* = 21.58, SD = 4.63). This pattern was consistent across teachers, with Treatment-group means exceeding Control-group means for each teacher (Table 7).

**Table 7:** Descriptive statistics for posttest mathematics achievement by group and teacher.

Group	Teacher	*M*	SD	*n*
Treatment	1	25.26	4.55	31
Treatment	2	28.13	5.21	38
Treatment	3	27.04	4.70	27
Treatment	4	26.23	4.86	31
Treatment	Total	26.73	4.93	127
Control	1	21.76	4.42	33
Control	2	22.26	4.00	34
Control	3	22.24	5.42	25
Control	4	20.31	4.73	36
Control	Total	21.58	4.63	128

After adjusting for pretest mathematics raw score, instructional group remained a statistically significant predictor of posttest mathematics achievement, *F*(1, 3.02) = 39.68, *p* = 0.008. Estimated marginal means showed that students in the Treatment group had significantly higher adjusted posttest scores [*M* = 26.66, SE = 0.36, 95% CI (25.96, 27.36)] than students in the Control group [*M* = 21.66, SE = 0.36, 95% CI (20.96, 22.36)]. The adjusted mean difference was 5.00 points, 95% CI [4.01, 5.99], *p* < 0.001 (Table 8).

**Table 8:** Teacher-adjusted ANCOVA results for posttest mathematics achievement.

Source	df	*F*	*p*
Pretest mathematics raw score	1, 246	102.60	< 0.001
Group (treatment–control)	1, 3.02	39.68	0.008
Math teacher	3, 3.00	2.04	0.287
Group × teacher	3, 246	2.49	0.061

The teacher main effect was not statistically significant under the random-effects test, *F*(3, 3.00) = 2.04, *p* = 0.287, indicating that posttest achievement did not differ significantly across teachers after accounting for baseline achievement and instructional group. The Group × Teacher interaction approached, but did not reach, statistical significance, *F*(3, 246) = 2.49, *p* = 0.061. This suggests that the magnitude of the intervention effect was generally consistent across teachers (Table 8).

**Figure 2:**
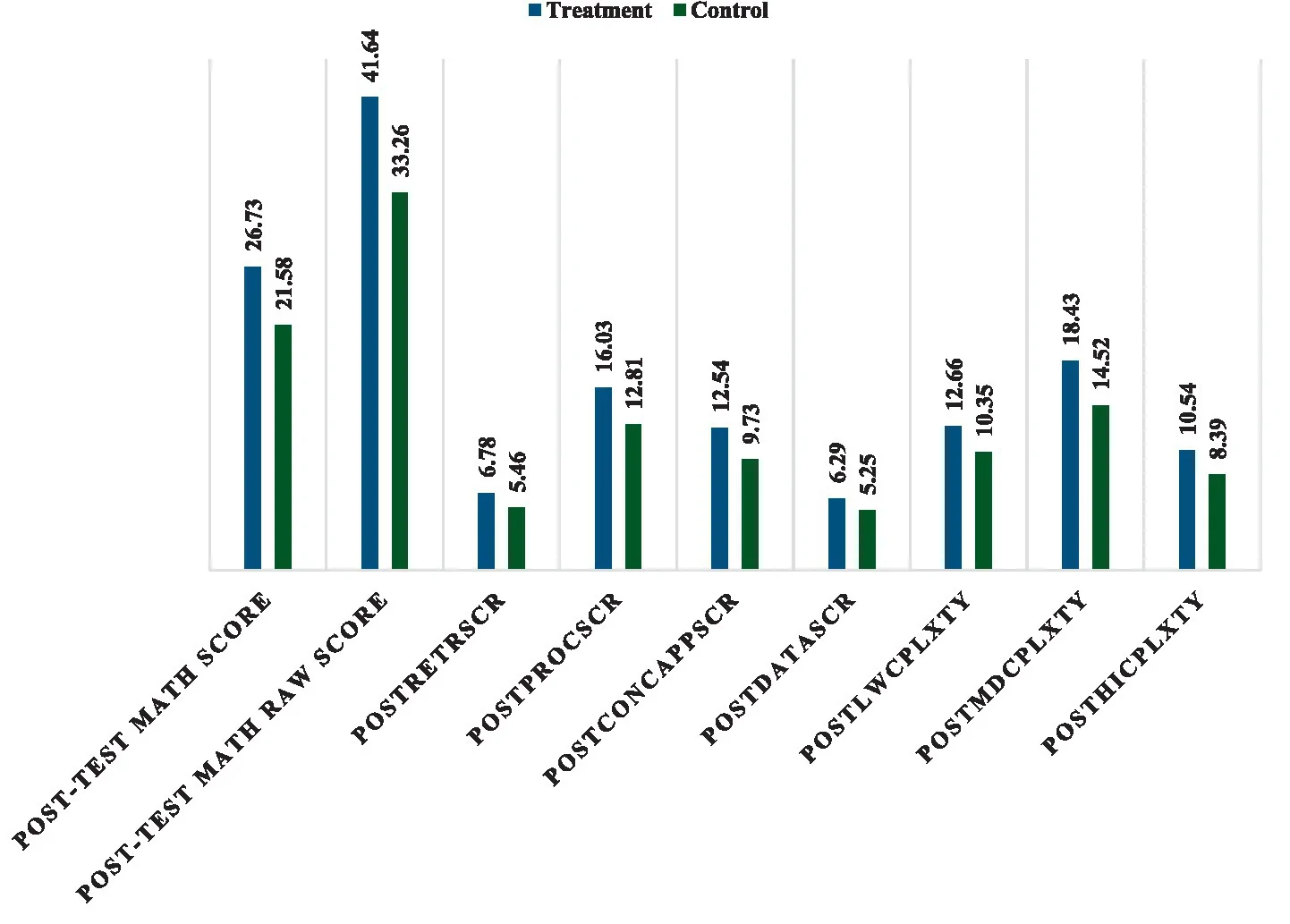
Post-intervention mathematics outcomes by experimental condition.

Taken together, the teacher-adjusted sensitivity analysis supported the robustness of the primary ANCOVA results. The intervention effect remained statistically significant after accounting for teacher-level clustering and the Group × Teacher interaction, indicating that the observed treatment advantage was not attributable to a single teacher or classroom context (Tables 9, 10, Figure 2).

**Table 9:** Adjusted posttest mathematics achievement by group.

Group	Adjusted *M*	SE	95% CI
Treatment	26.66	0.36	[25.96, 27.36]
Control	21.66	0.36	[20.96, 22.36]

Adjusted means were evaluated at the grand mean of pretest mathematics raw score (*M* = 34.17).

**Table 10:** Pairwise comparison of adjusted group means.

Comparison	Mean difference	SE	95% CI	*p*
Treatment−control	5.00	0.50	[4.01, 5.99]	< 0.001

Bonferroni adjustment was applied to pairwise comparisons.

#### 5.2.4 Cognitive process and complexity-specific outcomes

To examine whether the intervention was associated with improvement in theoretically relevant cognitive-performance domains, additional ANCOVA models were conducted for retrieval, procedural processing, conceptual application, data interpretation, and item-complexity performance. In each model, the corresponding pretest score was entered as a covariate, instructional group was entered as the fixed factor, and the posttest score served as the dependent variable. This approach evaluated whether students in the Treatment group outperformed students in the Control group on post-intervention cognitive-performance indicators after adjusting for baseline performance in the same domain.

As shown in Table 11, instructional group significantly predicted all posttest cognitive-performance outcomes after controlling for corresponding pretest scores. Students in the Treatment group demonstrated significantly higher adjusted posttest performance than students in the Control group on retrieval, *F*(1, 252) = 66.06, p < 0.001, partial η^2^ = 0.208; procedural processing, *F*(1, 252) = 80.75, *p* < 0.001, partial η^2^ = 0.243; conceptual application, *F*(1, 252) = 78.29, p < 0.001, partial η^2^ = 0.237; and data interpretation, *F*(1, 252) = 51.74, *p* < 0.001, partial η^2^ = 0.170. These findings indicate that the intervention effect was not limited to overall mathematics achievement but extended across specific cognitive-performance domains aligned with the instructional design.

**Table 11:** ANCOVA results for cognitive-performance outcomes.

Outcome	Covariate	Treatment adjusted *M* [95% CI]	Control adjusted *M* [95% CI]	Mean difference	F(*1*, 252)	*p*	Partial η^2^
Retrieval	Pre-retrieval	6.79 [6.56, 7.02]	5.45 [5.22, 5.68]	1.34	66.06	< 0.001	0.208
Procedural processing	Pre-procedural	16.02 [15.52, 16.52]	12.82 [12.33, 13.32]	3.20	80.75	< 0.001	0.243
Conceptual application	Pre-conceptual application	12.50 [12.07, 12.93]	9.77 [9.34, 10.20]	2.73	78.29	< 0.001	0.237
Data interpretation	Pre-data interpretation	6.30 [6.10, 6.51]	5.24 [5.04, 5.45]	1.06	51.74	< 0.001	0.170

Adjusted means were estimated at the grand mean of the corresponding pretest covariate. Treatment group *n* = 127; control group *n* = 128.

Additional ANCOVA models examined whether the intervention was associated with posttest performance across low-, medium-, and high-complexity mathematics items. As shown in Table 12, instructional group significantly predicted posttest performance at all three complexity levels after controlling for corresponding pretest complexity scores. Treatment students outperformed Control students on low-complexity items, *F*(1, 252) = 61.50, *p* < 0.001, partial η^2^ = 0.196; medium-complexity items, *F*(1, 252) = 86.63, *p* < 0.001, partial η^2^ = 0.256; and high-complexity items, *F*(1, 252) = 66.48, *p* < 0.001, partial η^2^ = 0.209. The strongest effect was observed for medium-complexity performance, although all three effects were substantial.

**Table 12:** ANCOVA results for complexity-specific mathematics outcomes.

Outcome	Covariate	Treatment adjusted *M* [95% CI]	Control adjusted *M* [95% CI]	Mean difference	F(*1*, 252)	*p*	Partial η^2^
Low complexity	Pre-low complexity	12.69 [12.27, 13.11]	10.33 [9.91, 10.74]	2.36	61.50	< 0.001	0.196
Medium complexity	Pre-medium complexity	18.41 [17.83, 18.99]	14.54 [13.96, 15.12]	3.87	86.63	< 0.001	0.256
High complexity	Pre-high complexity	10.46 [10.12, 10.80]	8.48 [8.14, 8.81]	1.98	66.48	< 0.001	0.209

Adjusted means were estimated at the grand mean of the corresponding pretest covariate. Treatment group *n* = 127; control group *n* = 128.

Taken together, these analyses provide convergent evidence that the intervention improved not only overall mathematics achievement but also performance across cognitive subdomains and item-complexity levels. The significant effects for retrieval, procedural processing, conceptual application, and data interpretation are consistent with the theoretical claim that the intervention supported multiple dimensions of cognitively grounded mathematical learning. Similarly, the significant effects across low-, medium-, and high-complexity items suggest that the intervention supported performance under varying levels of task demand rather than only improving performance on simpler items (Figure 2).

#### 5.2.5 Exploratory analysis: mindset and mathematics gain within the treatment group

An exploratory analysis examined the association between growth mindset and mathematics achievement gain within the Treatment group. Pearson correlation analysis indicated a moderate positive association between MindsetScore and MathGain, *r*(125) = 0.43, *p* < 0.001. Students reporting stronger growth-oriented beliefs tended to demonstrate larger mathematics gains over the intervention period.

Because mindset was measured only within the Treatment group, this finding should be interpreted as descriptive of individual differences in learning outcomes rather than as evidence of a moderating or causal effect. The observed association suggests that motivational beliefs may contribute to variability in achievement gains within cognitively grounded instructional environments and warrants further investigation in future research.

### 5.3 Integrated summary

Across all analyses, the results consistently supported the effectiveness of the cognitively grounded blended-learning intervention. Baseline equivalence analyses confirmed that Treatment and Control students entered the study with virtually identical levels of mathematics achievement, supporting the success of random assignment and strengthening the internal validity of subsequent comparisons. The primary ANCOVA demonstrated a substantial intervention effect on posttest mathematics achievement after controlling for baseline performance, with Treatment students achieving significantly higher adjusted posttest scores than Control students. Supplementary gain-score analyses yielded convergent findings, indicating that Treatment students experienced substantially greater mathematics achievement growth over the intervention period.

Importantly, the intervention effect remained statistically significant in the teacher-adjusted sensitivity analysis. After accounting for teacher-level clustering and potential variation across instructional contexts, students in the Treatment group continued to outperform students in the Control group by approximately five points on the posttest assessment. Neither the teacher main effect nor the Group × Teacher interaction reached statistical significance, suggesting that the intervention effect was generally consistent across participating teachers and was not driven by a single classroom or instructor.

The cognitive-performance analyses further extended these findings by demonstrating significant treatment advantages across multiple theoretically relevant domains, including retrieval, procedural processing, conceptual application, and data interpretation. Significant effects were also observed across low-, medium-, and high-complexity mathematics items. These results indicate that the intervention influenced not only overall mathematics achievement but also performance in domains aligned with the instructional emphasis on retrieval practice, procedural fluency, conceptual understanding, and application of knowledge under varying levels of task complexity.

Finally, exploratory analyses revealed a moderate positive association between growth mindset and mathematics achievement gains within the Treatment group. Although correlational and limited to students receiving the intervention, this finding suggests that motivational beliefs may contribute to individual differences in responsiveness to cognitively grounded instructional environments.

Taken together, the convergence of results across ANCOVA, gain-score analyses, teacher-adjusted sensitivity analyses, cognitive-performance outcomes, and exploratory mindset analyses provides a coherent pattern of evidence supporting the effectiveness of the intervention. The consistency of these findings across multiple analytic approaches strengthens confidence that the observed achievement advantages were attributable to the intervention rather than to baseline differences, teacher effects, or methodological artifacts.

### 5.4 Exploratory demographic sensitivity analyses

To examine whether the intervention effect varied across student demographic characteristics, a series of exploratory analyses of covariance (ANCOVAs) were conducted. In each model, posttest mathematics achievement served as the dependent variable, pretest mathematics achievement was included as the covariate, instructional group was entered as the primary factor, and a demographic variable (gender, race/ethnicity, parental level of education, lunch status, or living arrangement) was included together with its interaction with instructional group. These analyses were conducted to evaluate whether the effectiveness of the intervention differed across demographic subgroups after controlling for baseline mathematics achievement.

Across all models, pretest mathematics achievement remained a significant predictor of posttest performance, with partial η^2^ values ranging from 0.251 to 0.298, indicating that baseline achievement accounted for a substantial proportion of variance in posttest mathematics outcomes. More importantly, instructional group remained a statistically significant predictor of posttest achievement in every model. The adjusted treatment effect ranged from 5.17 to 5.79 points across analyses, and the effect size for instructional group remained consistently large (partial η^2^ = 0.189–0.285), demonstrating the robustness of the intervention effect after accounting for demographic variation.

The gender moderation analysis indicated that instructional group remained a significant predictor of posttest achievement, *F*(1, 250) = 99.84, *p* < 0.001, ηp^2^ = 0.285. The main effect of gender was not significant, *F*(1, 250) = 1.65, *p* = 0.200, ηp^2^ = 0.007, and the Group × Gender interaction was also not significant, *F*(1, 250) = 0.19, *p* = 0.664, ηp^2^ = 0.001. These findings indicate that the intervention produced comparable achievement benefits for female and male students.

Similarly, the race/ethnicity analysis demonstrated a significant intervention effect, *F*(1, 244) = 80.51, p < 0.001, ηp^2^ = 0.248. Although a significant main effect of race/ethnicity was observed, *F*(4, 244) = 6.59, *p* < 0.001, ηp^2^ = 0.097, indicating differences in adjusted achievement levels among racial/ethnic groups, the Group × Race/Ethnicity interaction was not significant, *F*(4, 244) = 1.21, *p* = 0.308, ηp^2^ = 0.019. Thus, while achievement levels differed somewhat across racial/ethnic groups, the magnitude of the intervention effect remained consistent.

The parental education analysis yielded a significant intervention effect, *F*(1, 246) = 73.78, p < 0.001, ηp^2^ = 0.231. Neither the main effect of parental education, *F*(3, 246) = 1.32, *p* = 0.270, ηp^2^ = 0.016, nor the Group × Parental Education interaction, *F*(3, 246) = 1.10, *p* = 0.348, ηp^2^ = 0.013, reached statistical significance. These findings suggest that intervention effectiveness did not depend on parental educational attainment.

The lunch-status analysis likewise demonstrated a significant intervention effect, *F*(1, 250) = 58.21, *p* < 0.001, ηp^2^ = 0.189. Neither the main effect of lunch status, *F*(1, 250) = 1.67, *p* = 0.198, ηp^2^ = 0.007, nor the Group × Lunch Status interaction, *F*(1, 250) = 0.45, *p* = 0.505, ηp^2^ = 0.002, was statistically significant. Consequently, the intervention appeared equally effective for students receiving free or reduced-price lunch and students receiving standard lunch services.

Finally, the living-arrangement analysis revealed a significant intervention effect, *F*(1, 246) = 86.24, *p* < 0.001, ηp^2^ = 0.260. Neither the main effect of living arrangement, *F*(3, 246) = 1.45, *p* = 0.228, ηp^2^ = 0.017, nor the Group × Living Arrangement interaction, *F*(3, 246) = 0.19, *p* = 0.902, ηp^2^ = 0.002, was statistically significant. Treatment students consistently outperformed Control students regardless of whether they lived with both parents, a single mother, a single father, or another guardian.

Taken together, the demographic sensitivity analyses revealed a highly consistent pattern of findings (Tables 13, 14). Across all demographic characteristics examined, instructional group remained a significant predictor of posttest mathematics achievement, whereas none of the interaction terms reached statistical significance. These results suggest that the intervention’s effectiveness was broadly stable across demographic subgroups and provide preliminary evidence that the achievement benefits observed in the present study were not limited to a particular gender, racial/ethnic group, socioeconomic subgroup, parental education level, or household structure. Given the exploratory nature of these analyses and the relatively small sample sizes within some demographic categories, these findings should be interpreted cautiously and replicated in larger multisite studies.

**Table 13:** Summary of exploratory demographic moderation analyses.

Moderator	Group Effect F(df)	*p*	ηp^2^	Interaction F(df)	*p*	ηp^2^
Gender	*F*(1, 250) = 99.84	< 0.001	0.285	*F*(1, 250) = 0.19	0.664	0.001
Race/ethnicity	*F*(1, 244) = 80.51	< 0.001	0.248	*F*(4, 244) = 1.21	0.308	0.019
Parental education	*F*(1, 246) = 73.78	< 0.001	0.231	*F*(3, 246) = 1.10	0.348	0.013
Lunch status	*F*(1, 250) = 58.21	< 0.001	0.189	*F*(1, 250) = 0.45	0.505	0.002
Living arrangement	*F*(1, 246) = 86.24	< 0.001	0.260	*F*(3, 246) = 0.19	0.902	0.002

Posttest mathematics achievement served as the dependent variable in all models, and pretest mathematics achievement was included as a covariate. Interaction terms tested whether the intervention effect differed across demographic subgroups.

**Table 14:** Adjusted posttest mathematics achievement means by demographic subgroup.

Moderator	Category	Treatment *M* (SE)	Control *M* (SE)
Gender	Female	26.95 (0.52)	22.01 (0.51)
	Male	26.51 (0.52)	21.12 (0.52)
Race/ethnicity	Asian	28.22 (0.93)	22.57 (0.91)
	Black/African American	25.32 (0.99)	17.88 (0.94)
	Hispanic/Latino	25.81 (0.69)	21.40 (0.70)
	Native American/Mixed	26.11 (1.49)	19.16 (1.77)
	White (Non-Hispanic)	27.30 (0.54)	22.80 (0.54)
Parental education	Associate’s degree	27.87 (0.97)	22.74 (0.92)
	Bachelor’s degree	26.06 (0.88)	22.00 (0.90)
	High school	26.35 (0.48)	21.32 (0.47)
	Master’s degree or higher	28.27 (1.10)	20.47 (1.24)
Lunch status	Free/reduced	26.35 (0.95)	20.39 (0.92)
	Standard	26.80 (0.40)	21.80 (0.40)
Living arrangement	Both parents	27.16 (0.65)	22.49 (0.67)
	Other	26.12 (1.03)	21.02 (0.90)
	Single parent (dad)	26.31 (0.88)	20.47 (0.85)
	Single parent (mom)	26.77 (0.59)	21.67 (0.62)

### 5.5 Associations between mathematics gain and fidelity indicators

Within the Treatment group, mathematics gain was positively associated with all fidelity indicators, suggesting that students exposed to higher levels of implementation quality tended to demonstrate larger achievement gains. The strongest association was observed between MathGain and the overall implementation fidelity index, *r* = 0.68, *p* < 0.001. Professional development fidelity was also strongly associated with MathGain, *r* = 0.61, *p* < 0.001. More moderate associations were observed for usage fidelity, *r* = 0.41, *p* < 0.001, and consultation fidelity, *r* = 0.35, *p* < 0.001. These correlations indicate that stronger intervention enactment was associated with greater mathematics gains, but they should not be interpreted as evidence that any single fidelity component independently caused achievement growth.

Because the fidelity indicators were conceptually related and empirically overlapping, the fidelity results were interpreted at the level of overall implementation quality rather than as separable causal pathways. In particular, professional development fidelity and the overall implementation fidelity index were very highly correlated, *r* = 0.95, indicating substantial redundancy between these measures. This overlap was expected because professional development fidelity was one of the largest components of the weighted implementation index and was theoretically central to successful intervention enactment. Accordingly, the individual fidelity indicators were not treated as statistically independent predictors of student achievement.

The high intercorrelation among fidelity indicators suggests that the intervention operated as an integrated instructional system in which teacher preparation, student engagement with the instructional platform, and consultation support functioned together as mutually reinforcing elements of enactment quality. Consequently, the fidelity findings are best interpreted as evidence of implementation sensitivity: students demonstrated larger mathematics gains when the intervention was enacted more fully and consistently. However, the correlational nature of the analyses precludes causal conclusions about the independent contribution of any single fidelity component.

Taken together, the fidelity findings support the interpretation that implementation quality was meaningfully associated with student outcomes. They are consistent with the theoretical expectation that cognitively grounded interventions depend on high-quality enactment to preserve core instructional features such as mastery-based progression, structured retrieval opportunities, adaptive sequencing, and targeted feedback. Nevertheless, because fidelity was not experimentally manipulated and the fidelity indicators were highly intercorrelated, these results should be interpreted as evidence of association between overall enactment quality and achievement gains rather than as evidence of causal effects of specific fidelity dimensions.

## 6 Discussion

### 6.1 Overview of findings and theoretical orientation

The present study examined the effectiveness of a cognitively grounded mathematics intervention implemented within a high school blended learning environment, with particular attention to achievement gains, implementation fidelity, and variability in outcomes across student subgroups. Results demonstrated a large and statistically robust effect of the intervention on mathematics achievement. Students in the Treatment group outperformed their Control peers by an average of 5.13 points on a statewide high-stakes assessment, corresponding to a large effect size (partial η^2^ = 0.281). This effect was accompanied by substantially reduced variability in gain scores relative to the Control group.

Together, these findings provide evidence that instruction grounded in cognitive science principles, when enacted within the blended learning environment examined in this study, was associated with meaningful and consistent learning gains in secondary mathematics. Although the results are encouraging, the findings should be interpreted within the context of a single-site pilot study and therefore require replication across additional educational settings. The discussion will interpret these outcomes through the lens of cognitive learning theory, with particular emphasis on mastery learning, retrieval-based practice, cognitive load management, and adaptive instructional design.

### 6.2 Effectiveness of the cognitively grounded intervention

The magnitude of observed achievement gains was substantial relative to many reported secondary mathematics interventions and aligns with outcomes documented in cognitively informed instructional models. However, because the study was conducted within a single educational context, caution is warranted when comparing the present results directly to broader populations or large-scale implementations. Students receiving the intervention demonstrated a mean gain of 6.04 points (SD = 1.55), compared to a mean gain of 0.91 points (SD = 6.69) for students in the Control group. Notably, the Treatment group exhibited a pronounced compression of gain-score variability, indicating that learning gains were broadly distributed across participants rather than concentrated among a small subset of high-performing students.

This pattern is consistent with theoretical accounts of cognitively grounded instruction. Mastery-based progression, retrieval-based practice, and spaced learning have been associated with improved learning outcomes in prior research (Bloom, 1984; Cepeda et al., 2006; Roediger and Karpicke, 2006; Rohrer and Taylor, 2007). Because these cognitive processes were not directly measured in the present study, the observed achievement gains should be interpreted as consistent with these theoretical mechanisms rather than as direct evidence that they occurred.

The reduction in gain-score variability may have implications for understanding how instructional structure influences student performance. One possible interpretation is that mastery-based progression and structured learning supports may help reduce performance variability among learners. However, because measures of cognitive load, fluid reasoning, working-memory capacity, and knowledge consolidation were not collected, the present study cannot directly determine the mechanisms responsible for the observed reduction in variability.

### 6.3 Cognitive mechanisms in a blended learning context

The blended learning environment served as an enabling structure for the implementation of cognitively grounded instructional principles rather than as an instructional mechanism in its own right. The online platform facilitated repeated retrieval opportunities, mastery-based progression, adaptive sequencing of practice, and systematic spacing of content, while face-to-face instruction provided opportunities for conceptual clarification, guided feedback, and knowledge consolidation. This integration is consistent with research suggesting that learning outcomes may depend more on instructional design features than on delivery modality alone (Anderson et al., 1995). The present study was designed according to these principles but did not directly assess the cognitive processes underlying student learning.

The cognitive-performance analyses provide additional support for this interpretation. After controlling for baseline performance, students in the Treatment group significantly outperformed students in the Control group across all assessed cognitive-performance domains, including retrieval, procedural processing, conceptual application, and data interpretation. Significant advantages were also observed across low-, medium-, and high-complexity mathematics items. Importantly, these effects extended beyond overall achievement scores, suggesting that the intervention was associated with improved performance across multiple mathematical domains. These outcome patterns are consistent with the instructional emphasis on retrieval practice, procedural fluency, conceptual understanding, and application of knowledge, although the underlying cognitive processes were not directly measured.

The reduced variability observed in Treatment-group gains further suggests that the blended instructional system functioned as a constraint-based learning environment, limiting opportunities for students to advance while important misconceptions remained unresolved. By requiring mastery before progression and providing targeted corrective practice, the intervention promoted more consistent learning trajectories across students. Such constraint-based designs are characteristic of cognitive tutoring and mastery-learning systems, which have repeatedly been shown to outperform conventional instruction in both average achievement and the consistency of learning outcomes across learners (Koedinger et al., 2012; Li et al., 2024; Meylani, 2024a).

### 6.4 Demographic variability as heterogeneity of response

Demographic analyses were conducted exclusively within the Treatment group to examine heterogeneity in response to the intervention rather than differences in access or exposure. This analytic choice isolates how the cognitively grounded instructional system functioned across student subgroups once the intervention was received.

Gender analyses revealed a statistically significant but modest difference in gains, with female students outperforming male students by 0.62 points (partial η^2^ = 0.040). Both groups demonstrated substantial gains, indicating that the intervention supported learning across genders. The magnitude of this difference aligns with prior research showing small gender differences in mathematics achievement under structured, mastery-oriented instructional conditions (Hyde et al., 2008).

Race and ethnicity analyses demonstrated significant variability in gain magnitude (partial η^2^ = 0.277). Asian students exhibited the largest gains (*M* = 7.56), while all other groups demonstrated average gains exceeding five points. Importantly, no group exhibited negligible or negative gains. These results indicate differential magnitudes of benefit rather than differential effectiveness and align with findings that prior opportunity structures and instructional alignment interact with cognitively demanding interventions (Ladson-Billings, 2006).

Socioeconomic indicators revealed a nuanced pattern. Parental education did not significantly moderate gains (*p* = 0.057), indicating robustness of the intervention across educational backgrounds. Lunch status demonstrated a modest but statistically significant effect (partial η^2^ = 0.057), with students receiving free or reduced lunch gaining an average of 1.04 points less than peers receiving standard lunch. Despite this difference, gains among economically disadvantaged students remained substantial (*M* = 5.16), supporting the conclusion that the intervention functioned effectively across socioeconomic strata while acknowledging persistent contextual constraints.

Living arrangement did not significantly moderate achievement gains (*p* = 0.261), providing additional evidence that the intervention buffered against variability associated with family structure.

These subgroup analyses do not indicate differential program effectiveness or deficit-based explanations of learning outcomes. Instead, they describe heterogeneity of response within a cognitively constrained instructional environment in which all demographic groups demonstrated substantial positive gains relative to baseline.

Although positive achievement gains were observed across all demographic groups represented in the sample, the demographic findings should be interpreted cautiously. The study was conducted within a single school, and several subgroup sample sizes were relatively small and unequal. Consequently, these analyses are best viewed as exploratory indicators of heterogeneity of response rather than definitive evidence regarding demographic generalizability.

### 6.5 Implementation fidelity and learning outcomes

Associations between mathematics gain and fidelity indicators revealed a clear pattern of implementation sensitivity. Implementation fidelity exhibited the strongest association with MathGain (*r* = 0.68), followed closely by professional development fidelity (*r* = 0.61). Usage fidelity (*r* = 0.41) and consultation fidelity (*r* = 0.35) demonstrated moderate associations with achievement gains.

These results are consistent with theoretical perspectives emphasizing the importance of high-quality instructional enactment for effective implementation. Professional development fidelity reflects teachers’ understanding of the cognitive model underlying the intervention, which directly shapes instructional decisions, feedback quality, and pacing (Meylani et al., 2025). The strong intercorrelation between professional development fidelity and implementation fidelity (*r* = 0.95) indicates that teacher preparation functioned as the primary driver of high-quality enactment.

The more moderate associations with usage and consultation fidelity reflect structural realities of blended learning environments. Time-on-task supports learning only when practice is cognitively productive, and consultation effectiveness depends on both teacher capacity and student engagement. The weighted composite fidelity measure captured these dynamics and explained substantial variance in learning outcomes, consistent with prior research emphasizing fidelity as a critical determinant of effectiveness in cognitively complex interventions (O’Donnell, 2008).

### 6.6 Implications for cognitive and psychological theories and instructional design

The present findings provide evidence that instruction grounded in cognitive science principles can yield robust mathematics learning gains when implemented with high fidelity within the educational context examined in this study. Replication across multiple schools, grade levels, and instructional settings will be necessary to determine the extent to which these findings generalize beyond the present sample. However, the study was designed to evaluate educational outcomes rather than directly test the cognitive mechanisms proposed by the underlying theoretical framework. These results reinforce central claims of cognitive load theory, which emphasizes that learning outcomes depend on reducing extraneous processing demands and aligning instructional design with the limits and structure of human working memory (e.g., Paas et al., 2003; Sweller, 1988). Cognitive load theory proposes that instructional designs which effectively manage intrinsic, extraneous, and germane load may support schema development and knowledge transfer. The achievement patterns observed in the present study are consistent with these theoretical predictions; however, cognitive load and schema acquisition were not directly measured.

Beyond cognitive architecture, the findings also inform psychological understanding of motivation–cognition interactions in mathematics learning. Growth mindset theory posits that students’ beliefs about the malleability of intelligence relate systematically to engagement, persistence, and responses to challenge (Dweck and Leggett, 1988; Yeager and Dweck, 2020). Empirical research further indicates that growth-oriented beliefs are associated with deeper engagement and sustained effort in cognitively demanding domains such as mathematics, and that these motivational orientations covary with academic achievement (Dong et al., 2023; Stohlmann and Yang, 2024).

Within the Treatment group, growth mindset demonstrated a moderate positive association with mathematics achievement gain (*r* = 0.43). Students reporting stronger growth-oriented beliefs tended to exhibit larger gains over the intervention period. Although the correlational nature of the finding precludes causal interpretation, the result is consistent with literature suggesting that motivational beliefs and cognitive processes jointly influence academic learning.

Importantly, this association emerged in a context in which all students showed substantial improvement, supporting the interpretation that mindset indexed residual individual variability in learning outcomes rather than functioning as a determinant of instructional effectiveness. Because mindset was measured only within the Treatment group, this relation is interpreted as descriptive of within-condition variability rather than as evidence of moderation or differential treatment effects across instructional conditions. No causal inference is implied by this association.

The observed association between mindset and learning outcomes aligns with emerging research on the interplay between motivational processes and cognitive load. Recent work indicates that instructional approaches that reduce cognitive load are also associated with improvements in motivation and engagement, underscoring the interdependence of cognitive and motivational processes in academic learning (Evans et al., 2024). When instructional design effectively manages cognitive constraints, students’ beliefs about their capacity to develop ability align with cognitive supports, contributing to individual differences in learning gains. Under this account, motivational beliefs do not replace cognitive mechanisms but operate alongside them, shaping variability around a shared baseline of effective instruction.

Taken together, these findings underscore that durable learning outcomes reflect the interaction of cognitive architecture, instructional structure, and motivational beliefs rather than modality, exposure, or engagement alone. The results support integrative theoretical frameworks that incorporate both cognitive and motivational constructs in models of academic learning, particularly in cognitively demanding domains such as secondary mathematics.

## 7 Conclusion

### 7.1 Summary of key findings

This randomized pilot study evaluated a cognitively grounded mathematics intervention implemented within a high school blended learning environment. The primary ANCOVA demonstrated a large and statistically robust intervention effect on posttest mathematics achievement after controlling for baseline performance. Supplementary gain-score analyses converged with this finding, showing that Treatment group students demonstrated substantially greater mathematics growth than Control group students. Baseline equivalence analyses confirmed that the two groups entered the study with comparable pre-intervention mathematics achievement, strengthening confidence that the observed post-intervention differences were attributable to the instructional condition within the study context.

In addition to producing higher mean achievement outcomes, the Treatment group showed reduced variability in gain scores relative to the Control group. This pattern suggests that learning gains were more consistently distributed among students receiving the intervention. However, because the study did not directly measure cognitive load, retrieval success, working-memory demands, or schema acquisition, this reduction in variability should be interpreted as an educational outcome consistent with the intervention’s theoretical rationale rather than as direct evidence of specific cognitive mechanisms.

Exploratory demographic sensitivity analyses indicated that the intervention effect was generally consistent across the demographic characteristics examined. Although achievement levels and gain magnitudes varied somewhat across subgroups, Treatment students consistently outperformed Control students, and no significant Group × Demographic interaction was observed. These findings provide preliminary evidence that the intervention benefits were not limited to a single demographic subgroup. However, given the pilot nature of the study, unequal subgroup sizes, and single-site design, these results should be interpreted cautiously and replicated in larger multisite studies.

Implementation fidelity was positively associated with mathematics gain within the Treatment group, with the strongest association observed for the overall implementation fidelity index. Professional development fidelity also showed a strong positive association with mathematics gain. These findings suggest that stronger overall enactment of the intervention was associated with larger student achievement gains. Because fidelity indicators were highly intercorrelated and fidelity was not experimentally manipulated, these associations should be interpreted as evidence of implementation sensitivity rather than as evidence for the independent causal influence of specific fidelity components.

Overall, the findings suggest that mastery-based, cognitively grounded instructional approaches warrant further investigation as promising strategies for improving secondary mathematics achievement. Broader claims regarding effectiveness across diverse educational contexts, grade levels, and implementation conditions require replication through larger multisite studies.

### 7.2 Merits and novelties of the study

The present study does not claim novelty in the individual theories, instructional principles, or technological tools on which the intervention was based. Cognitive load theory, mastery learning, retrieval-based practice, adaptive sequencing, blended learning, and growth mindset have all been examined extensively in prior educational and psychological research. Accordingly, the contribution of this study does not rest on introducing new constructs, but on integrating established principles into a coherent instructional model and evaluating that model in an authentic high school mathematics setting using a randomized pilot design.

The primary contribution lies in the operationalization of cognitive learning principles within a year-long blended mathematics intervention. Rather than treating blended learning as a modality-based reform, the study examined an instructional system designed around mastery-based progression, structured retrieval opportunities, adaptive sequencing, and targeted feedback. The results provide evidence that this theoretically grounded instructional model was associated with substantial improvements in mathematics achievement. However, because cognitive mechanisms were not directly measured, the findings should be understood as outcome-level evidence consistent with cognitive learning theory rather than direct evidence that specific mechanisms such as reduced cognitive load or schema acquisition mediated the observed effects.

A second contribution concerns the interpretation of blended learning. The study positions the blended environment as an enabling infrastructure for structured practice, progress monitoring, feedback, and mastery-based sequencing rather than as an instructional mechanism in itself. This distinction contributes conceptual clarity to a literature in which technology use is sometimes treated as the active ingredient of instructional improvement. In the present study, the educational value of the blended environment appeared to depend on the organization and enactment of instruction, not on technology use alone.

Third, the study contributes to implementation research by modeling fidelity as a proportional, capped, and theoretically weighted index of enactment quality. This approach moved beyond a simple participation-versus-nonparticipation measure and allowed implementation quality to be examined as a continuous feature of the intervention. At the same time, the high intercorrelation among fidelity indicators indicates that the components should be interpreted as overlapping aspects of a broader implementation-quality construct rather than as independent causal predictors. This more cautious interpretation strengthens the methodological transparency of the study.

Finally, the study contributes preliminary evidence regarding the consistency of intervention effects across student subgroups. Exploratory demographic analyses suggested that the treatment advantage was broadly stable across gender, race/ethnicity, parental education, lunch status, and living arrangement. These findings are promising from an equity-oriented perspective, but they should not be interpreted as definitive evidence of demographic generalizability. Larger multisite studies with balanced subgroup samples are needed to evaluate whether the observed pattern holds across broader populations and educational contexts.

### 7.3 Limitations

Several limitations should be considered when interpreting the findings. First, the study was conducted within a single high school, which limits the generalizability of the results to other schools, districts, grade levels, and instructional contexts. Although the randomized design strengthens internal validity and supports causal inference within the study setting, replication across multiple educational environments is necessary before broader conclusions regarding generalizability can be drawn.

Second, the demographic subgroup analyses were exploratory and involved unequal sample sizes across categories. While these analyses provided useful preliminary insights regarding the consistency of intervention effects across student groups, they should not be interpreted as definitive evidence of demographic generalizability. Future studies with larger and more balanced subgroup samples are needed to evaluate potential differences in responsiveness across populations more rigorously.

Third, the implementation fidelity indicators were highly intercorrelated, particularly professional development fidelity and the overall implementation fidelity index. This substantial overlap limited the ability to isolate the unique contribution of individual fidelity components and suggests that the indicators reflected interconnected aspects of a broader implementation-quality construct. Moreover, fidelity was examined using correlational analyses rather than experimental manipulation. Consequently, the fidelity findings are best interpreted as evidence that stronger overall implementation quality was associated with improved student outcomes rather than as evidence of the independent causal influence of any specific fidelity component.

Fourth, the study evaluated achievement outcomes using pretest and posttest mathematics assessments, which capture academic performance but do not directly assess the cognitive processes targeted by the intervention. Although the instructional model was designed around principles derived from cognitive load theory, retrieval-based learning, and mastery learning, no direct measures of cognitive load, working-memory demands, retrieval success, schema development, or related cognitive constructs were collected. As a result, the findings demonstrate the effectiveness of the instructional model as an educational intervention but cannot directly verify the specific cognitive mechanisms responsible for the observed achievement gains. Future research should incorporate direct cognitive measures to evaluate these theoretical mechanisms more rigorously.

Fifth, mindset was assessed only within the Treatment group and examined using correlational analyses. Accordingly, conclusions regarding the role of mindset should be interpreted cautiously and cannot establish causal, predictive, or moderating relationships.

Sixth, students were nested within teachers and instructional contexts, yet the present pilot study did not estimate multilevel models or cluster-adjusted standard errors. Although supplementary teacher-adjusted analyses indicated that the intervention effect remained robust across instructional contexts, future large-scale investigations should explicitly model classroom- and teacher-level clustering to evaluate the stability of intervention effects within hierarchical data structures.

Finally, the study evaluated outcomes over a single academic year. The extent to which the observed achievement gains persist over time, transfer to subsequent mathematics coursework, or influence longer-term academic trajectories remains unknown and warrants future investigation.

Despite these limitations, the randomized controlled design, strong baseline equivalence, substantial effect sizes, convergent findings across multiple analytic approaches, and consistent associations between implementation quality and student outcomes provide considerable confidence in the central conclusion that the cognitively grounded blended learning intervention was associated with meaningful improvements in secondary mathematics achievement.

### 7.4 Implications for policy and practice

The findings support several actionable implications for educational policy and instructional practice. Investment in cognitively grounded instructional systems yields substantial returns in student learning when such systems are implemented with fidelity. Professional development emerges as a critical lever for effectiveness, underscoring the need for sustained teacher preparation focused on cognitive principles rather than procedural compliance. Blended learning initiatives benefit from prioritizing instructional design quality over technology adoption, as learning outcomes depend on cognitive alignment rather than delivery mode.

These implications also align with broader policy frameworks connecting blended learning to equitable, high-quality education. In particular, blended learning models may support SDG 4 when they are designed to improve academic outcomes, strengthen learner agency, support non-cognitive development, and expand access to flexible, inclusive learning opportunities (Meylani, 2026). From this perspective, the present findings contribute to the policy argument that blended learning should be evaluated not by technology adoption alone, but by the extent to which it advances meaningful, measurable, and equitable learning outcomes.

At the school and district levels, the results support adoption of mastery-based, cognitively grounded interventions as viable strategies for improving secondary mathematics outcomes across diverse student populations.

### 7.5 Directions for future research

Future research should extend this work through multi-site randomized trials to examine generalizability across contexts. Longitudinal studies should investigate retention and transfer of learning beyond a single academic year. Experimental manipulation of fidelity components would clarify causal mechanisms underlying implementation sensitivity. Integration of process-level measures, such as retrieval success rates and error patterns, would further strengthen links between cognitive theory and observed achievement outcomes.

### 7.6 Concluding remarks

This study demonstrates that a cognitively grounded mathematics intervention implemented in a blended high school environment produces large, consistent, and educationally meaningful achievement gains. The results reinforce foundational principles of cognitive learning theory and provide clear evidence that instructional effectiveness depends on both theoretical grounding and high-quality implementation. Overall, the findings demonstrate that instructional systems explicitly aligned with human cognitive architecture yield large, consistent, and theoretically interpretable learning gains, reinforcing the central role of cognitive mechanisms in explaining educational outcomes.

## Data Availability

The original contributions presented in the study are included in the article/Supplementary material, further inquiries can be directed to the corresponding author.
