# Supplementary material for: Effectiveness of a cognitively grounded mathematics intervention in a high school blended learning environment: achievement gains, implementation fidelity, and demographic variability
Source: Front Psychol. 2026 Aug 6;17:1800998. doi: 10.3389/fpsyg.2026.1800998 (PMC13491030; doi:10.3389/fpsyg.2026.1800998)
Supplement: Supplementary file 1 [file Supplementary_File_1.pdf]

Appendix A

Representative Paired Lesson and Fidelity Mapping Illustrating Instructional Contrast**

This appendix provides a representative, process-level illustration of how the cognitively grounded intervention differed from business-as-usual mathematics instruction. The paired lesson comparison specifies learner actions, instructional constraints, use of technology, and teacher roles for identical mathematical content delivered within the same instructional timeframe. The appendix also maps each lesson component to the fidelity indicators used in the study, clarifying how fidelity indexed enactment of theoretically specified cognitive mechanisms rather than general compliance or instructional effort.

A.1 Lesson Context

- Mathematical Topic: Solving Linear Equations with Variables on Both Sides
- Grade Level: 11
- Instructional Duration: One 50-minute class period
- Content Objective: Solve linear equations in one variable and justify solution steps
- Instructional Setting: High school mathematics classroom with blended learning infrastructure

Both instructional conditions addressed the same content objective during the same point in the curriculum sequence.

A.2 Cognitively Grounded Intervention Lesson

A.2.1 Instructional Objective

Students solve linear equations with variables on both sides and demonstrate procedural fluency and conceptual justification through successful retrieval, mastery-based progression, and error-corrected practice.

A.2.2 Technological Environment

Students work on individual digital devices using an instructional platform that:

- delivers retrieval-based tasks,
- records responses in real time,
- enforces mastery thresholds before progression,
- adapts task sequencing based on accuracy and error patterns, and
- provides instructors with a live diagnostic dashboard displaying student performance and progression status.

Technology functions as a constraint system that regulates instructional progression and preserves cognitive design features across classrooms.

A.2.3 Lesson Sequence

1. Retrieval-Based Prerequisite Check (10 minutes)

- Students begin with a short retrieval task targeting prerequisite knowledge (inverse operations, combining like terms).
- The platform presents five short-response problems without worked examples or hints.
- Students submit responses individually.
- Advancement requires meeting a predefined mastery threshold.
- Students not meeting the threshold are automatically assigned to a corrective sequence consisting of worked examples followed by guided problem completion.
- Access to the main lesson sequence remains restricted until prerequisite mastery is demonstrated.

Cognitive function:

Stabilizes prerequisite schemas and constrains working-memory demands during subsequent problem solving.

2. Guided Schema Construction (10 minutes)

- Students receive a worked example illustrating variable isolation on both sides of an equation.
- The worked example highlights structural features such as maintaining equivalence and balancing operations.
- The platform presents a partially completed problem requiring students to generate missing solution steps.
- Immediate feedback follows each response.
- Errors trigger step-level guidance rather than full solution disclosure.

Cognitive function:

Supports schema acquisition while reducing extraneous processing demands.

3. Retrieval-Oriented Practice with Adaptive Sequencing (20 minutes)

- Students complete a sequence of problems requiring full solution generation.
- Problem types are interleaved with previously learned equation formats.
- Task sequencing adapts based on accuracy and diagnosed misconceptions.
- Incorrect responses trigger targeted corrective practice aligned with the specific error pattern.
- Students repeat retrieval attempts until mastery criteria are met.
- Progression to new problem types remains contingent on demonstrated understanding.

Cognitive function:

Promotes consolidation through effortful retrieval and prevents premature advancement.

4. Teacher Role and In-Person Interaction (Throughout)

- The teacher monitors student progress through the real-time dashboard.
- Teacher interactions focus on diagnosing misconceptions and delivering targeted feedback.
- Whole-class explanation is minimized.
- Instructional decisions reflect learner performance rather than fixed pacing.

Teacher function:

Acts as diagnostician and cognitive coach rather than primary content deliverer.

A.2.4 Lesson Closure

- Students who meet mastery criteria complete a brief reflective prompt explaining solution strategies.
- Students not meeting mastery criteria continue corrective practice in subsequent sessions.

A.3 Business-as-Usual Lesson

A.3.1 Instructional Objective

Students learn procedures for solving linear equations with variables on both sides.

A.3.2 Technological Environment

Technology use varies by classroom and typically includes presentation slides or optional digital worksheets. No adaptive sequencing, mastery enforcement, or real-time diagnostics are implemented.

A.3.3 Lesson Sequence

1. Whole-Class Explanation (15 minutes)

- The teacher introduces the topic through verbal explanation and board-based demonstrations.
- Students observe worked examples and take notes.
- Instruction follows a predetermined pacing schedule.
- No formal assessment of prerequisite knowledge occurs prior to instruction.

Cognitive condition:

Places simultaneous demands on working memory during explanation and note-taking.

2. Guided Practice (15 minutes)

- The teacher works through sample problems with the class.
- Students respond orally or copy solutions.
- Errors are addressed selectively and inconsistently.

3. Independent Practice (15 minutes)

- Students complete a fixed set of practice problems during class or as homework.
- All students receive the same problems regardless of performance.
- Feedback is delayed until later review or grading.

Cognitive condition:

Allows progression without mastery and provides limited retrieval-based reinforcement.

4. Teacher Role

- The teacher manages pacing and classroom behavior.
- Instructional decisions follow the curriculum calendar.
- Individual misconceptions are addressed opportunistically.

A.3.4 Lesson Closure

- The class advances to the next scheduled topic regardless of individual understanding.

A.4 Mapping of Lesson Components to Fidelity Indicators

This section maps specific components of the cognitively grounded lesson to the fidelity indicators used in the study. The mapping clarifies how fidelity indexed enactment of theoretically specified cognitive mechanisms rather than general implementation compliance.

A.4.1 Implementation Fidelity

Indexed Lesson Components:

- Enforcement of prerequisite mastery prior to progression
- Adaptive sequencing of tasks based on learner performance
- Structured retrieval-based practice requiring solution generation
- Immediate, task-level feedback following responses
- Restriction of advancement in the presence of unresolved misconceptions

Cognitive Mechanism Indexed:

Reduction of extraneous cognitive load and stabilization of prerequisite schemas supporting retrieval from long-term memory.

A.4.2 Professional Development Fidelity

Indexed Lesson Components:

- Teacher use of dashboard data to diagnose misconceptions
- Targeted feedback aligned with specific error patterns
- Suppression of whole-class explanation when retrieval practice is indicated
- Instructional decisions based on learner state rather than instructional timing

Cognitive Mechanism Indexed:

Teacher-mediated regulation of cognitive load and alignment of instructional support with learner expertise.

A.4.3 Usage Fidelity

Indexed Lesson Components:

- Student completion of retrieval-based prerequisite checks
- Engagement with corrective practice following errors
- Participation in interleaved retrieval sequences
- Persistence until mastery thresholds are met

Cognitive Mechanism Indexed:

Repeated successful retrieval and consolidation of schemas in long-term memory.

A.4.4 Consultation Fidelity

Indexed Lesson Components:

- Teacher–student interactions focused on error diagnosis
- Alignment of in-person support with platform-generated performance data
- Reinforcement of mastery expectations and retrieval standards

Cognitive Mechanism Indexed:

Reduction of ineffective guidance and alignment of feedback with cognitive processing demands.

A.4.5 Technology as a Fidelity-Relevant Constraint System

Across all fidelity dimensions, technology functioned as a mechanism for enforcing instructional constraints rather than as a content delivery tool. Platform features—including mastery thresholds, adaptive sequencing, response logging, and real-time diagnostics—operationalized cognitive principles consistently across classrooms. Variation in fidelity therefore reflects differences in enactment of these constraints rather than differences in access to technological resources.

A.5 Summary

The paired lesson and fidelity mapping demonstrate how identical content objectives were enacted under distinct cognitive conditions. The cognitively grounded intervention constrained progression, enforced mastery, structured retrieval, and used technology to preserve instructional design integrity. Business-as-usual instruction emphasized exposure, fixed pacing, and variable feedback. Fidelity indicators indexed the degree to which theoretically specified cognitive mechanisms were preserved during enactment, providing a concrete foundation for interpreting achievement gains and fidelity–outcome associations reported in the main analyses.
